# Supplementary material for: Membrane-embedded polar residues target membrane proteins for degradation by the quality control protease FtsH
Source: Nat Commun. 2026 Feb 23;17:3067. doi: 10.1038/s41467-026-69829-8 (PMC13039401; doi:10.1038/s41467-026-69829-8)
Supplement: Supplementary file 1 — Supplementary Information [file 41467_2026_69829_MOESM1_ESM.pdf]

a

TM segment	Sequence	Predicted ΔG
1	LVKITRLFPVWALLLSVAAYF	0.318
2	VGPLLMLIMFAMGVT	1.415
3	APVAAATFLHYLIMPLTAWILAMLF	-0.246
3 (mutated)	APVAAATFLHYQIMPLTAWILAML	1.826
4	AGMVLVGSVASGTASNVMIYLA	3.305
5	ALSVTISAVSTLVGVFATPLLT	1.488
6	VVGMLKSILQIVVIPITAGLVIHHT	2.167
7	YLPAMSMVSILAIISAVVA	0.682
8	VVIIAVILHNGIGLLSGYWGGKLF	2.175
9	TSRTLAIEVGMQNSGLAATLG	4.627
10	AALPGALFSVWHNLSGSLLAGYW	3.658

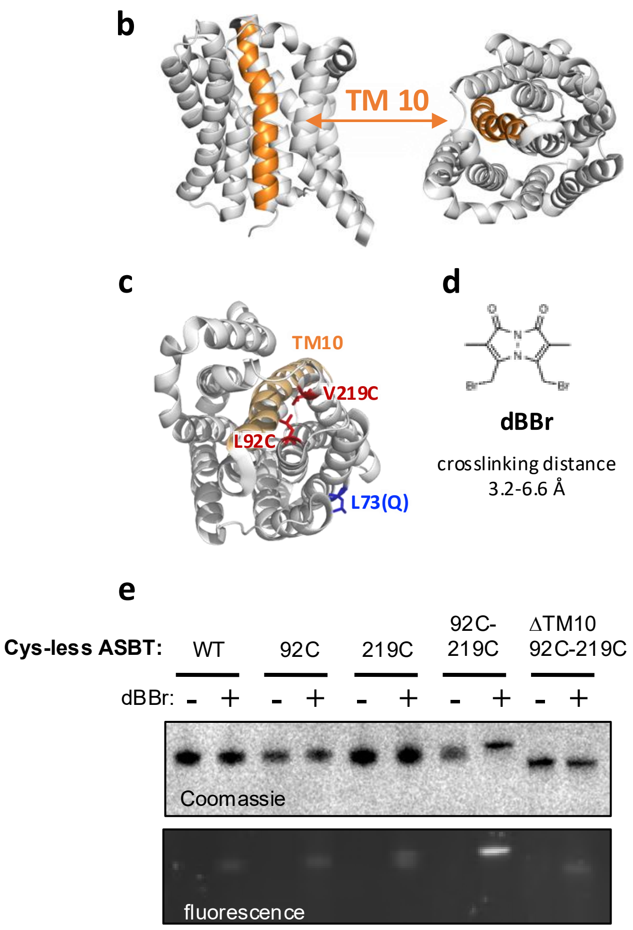

Supplementary figure 1. Probing the folding status of ASBT L73Q by Crosslinking.

a. The predicted biological hydrophobicity of ASBT TM segments, as obtained by inputting the TM segments from 4N7X to the ΔG predictor (Hessa et al. 2007 [87]). The mutated L73Q is highlighted. A negative ΔG indicates greater hydrophobicity, and TMs with ΔG < 2 are considered robustly inserted (Bernsel et al. 2008, doi: 10.1073/pnas.0711151105).

b. Crystal structure of ASBT (PDB: 4N7X), viewed from within (left) or above(right) the plane of the membrane. The central location of TM10 (orange) suggests that its deletion will likely cause misfolding.

c. Location of the two engineered Cys in the ASBT structure. The crosslinked residues at positions 92 and 219 are located at the edges of TMs 4 and 8, which form extensive interactions with TM3, where L73Q lies, and with TM10. Their proximity, as assayed by crosslinking, can serve as a measure for the proper folding of this structural module.

d. Structure of the crosslinker dBBr, having a crosslinking distance of 3.2-6.6 Å.

e. Crosslinking of ASBT by dBBr in native membranes confirms that two engineered cysteines are required for crosslinking. Crosslinking was demonstrated by a shift in SDS-PAGE migration (Coomassie) and by the appearance of a fluorescent band of crosslinked protein upon the reaction of a single dBBr with two cysteines simultaneously (fluorescence). The indicated ASBT variants were cross-linked in native membranes, followed by purification and SDS-PAGE.

a

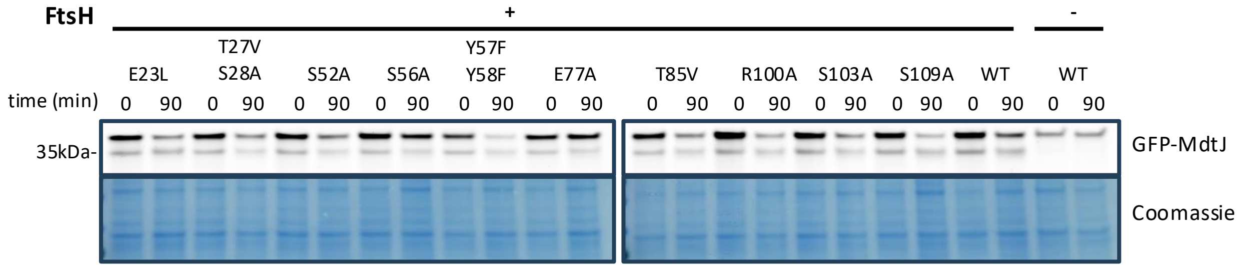

b

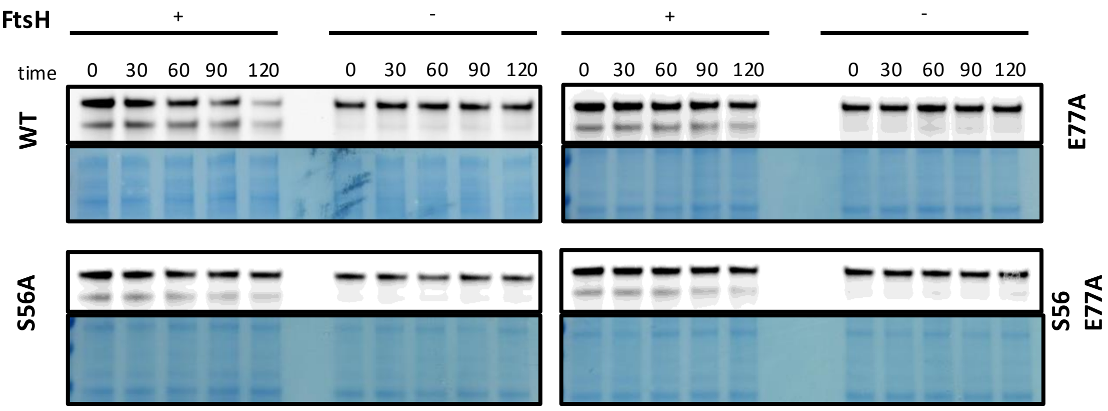

c

residue	TM	length	Sequence	Predicted ΔG
13-32	TM1	20	WIYLIIAILLEVIGTSVMKL	-0.121
44-65	TM2	22	IGYLLMLISIGLSYYMLALAAT	-0.855
44-65 Q mutant	TM2	22	IGYLLMLISIGQSYYMLALAAT	1.027
71-93	TM3	23	VAYAFWEGLGLILITLVSIFVID	-0.751
71-93 Q mutant	TM3	22	VAYAFWEGQGLILITLVSIFVID	0.468
96-114	TM4	19	MTITRLISLFALLSGAILI	0.009

d

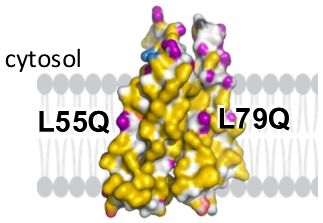

e

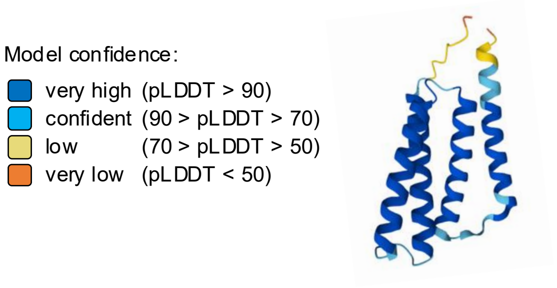

f

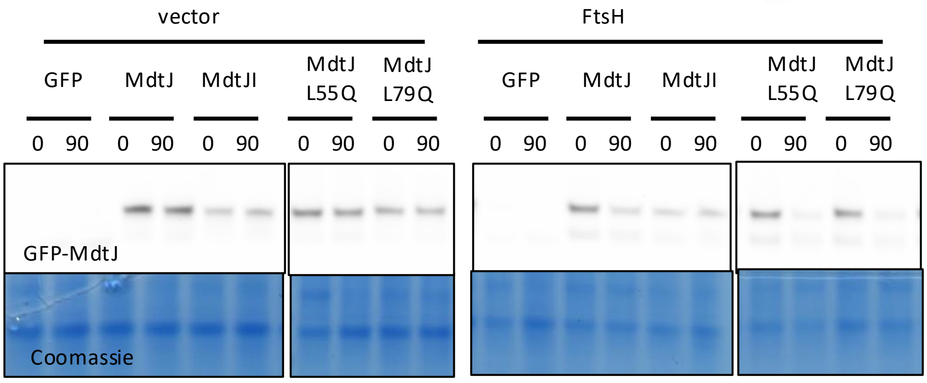

Supplementary figure 2. Representative raw data showing the effects of polar residues in MdtJ TMs (Fig 3).

(a) Effect of substituting polar residues of MdtJ with similarly sized apolar amino acids on degradation. Mutations to S56 and E77 perturb the degradation. Degradation of wild-type (WT) GFP-MdtJ in the presence or absence of FtsH are shown as controls.

(b) Degradation time course of GFP-MdtJ mutants in the presence and absence of FtsH, showing that the mutations E77L or S56A slow down the degradation, and their combination almost completely abolishes degradation.

(c) The predicted ΔG of insertion of MdJ TM segments before and after mutagenesis, as predicted by ΔG predictor (Hessa et al. 2007[83])

(d) The positions of the L55Q and L79Q mutations on the lipid-facing side of MdtJ. The hydrophilic groups of the glutamines are colored purple. Color coding is as in Fig. 3c.

(e) AlphaFold model of MdtJ. Confidence scoring according to AlphaFold, suggests that the prediction of the TM domain is accurate.

(f) Effect of L55Q and L79Q mutations on GFP-MdtJ degradation in the absence and presence of FtsH.

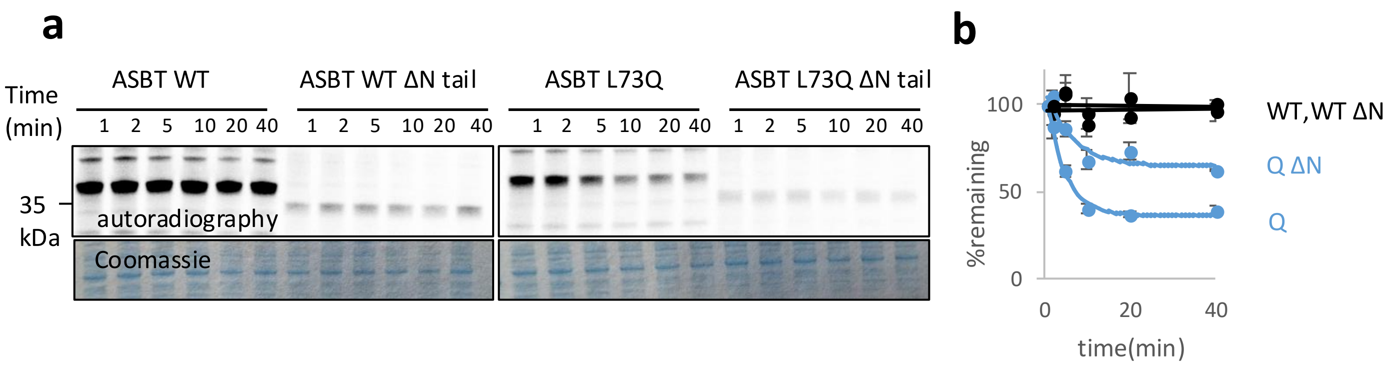

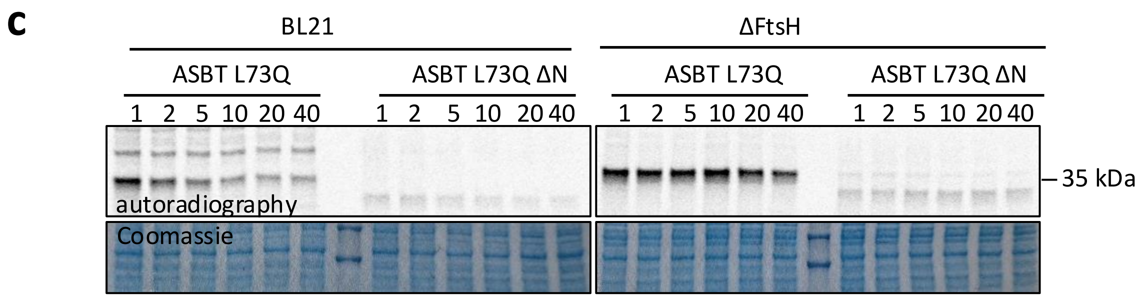

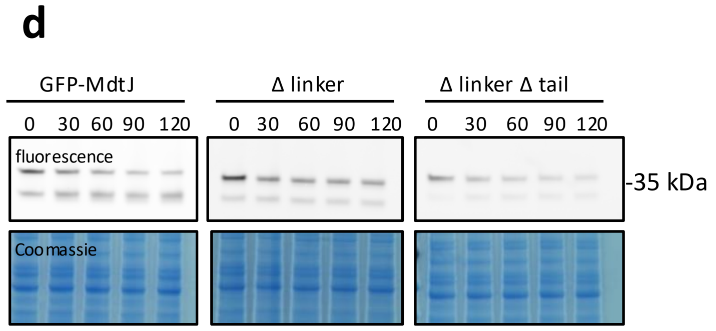

e

	Δlinker	Δtail
**original**	GITHGMDELYK MQPRQYTSQVK	HHGTVNISSNN
**mutated**	GSQVK	HHG

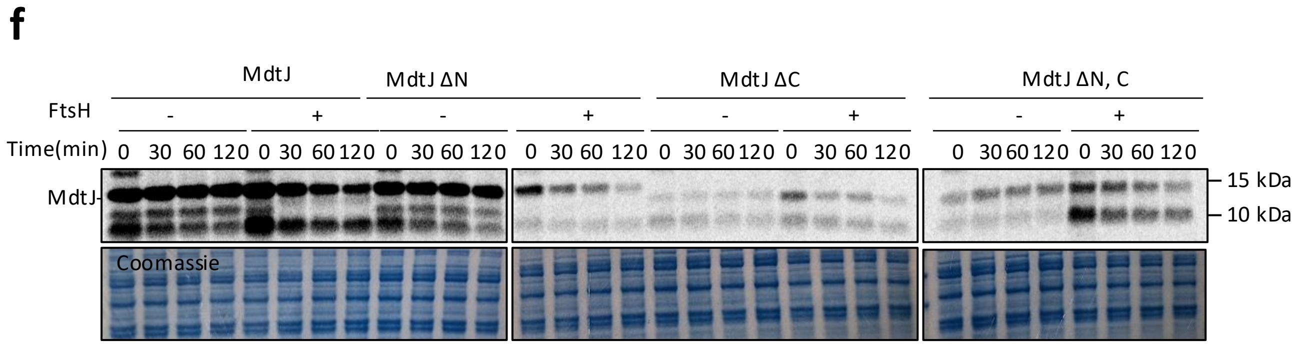

g

	Δloop	Δtail
**original**	MQPRQYTSQV KH	HHGTVNISS NN
**mutated**	MQPRH	HH

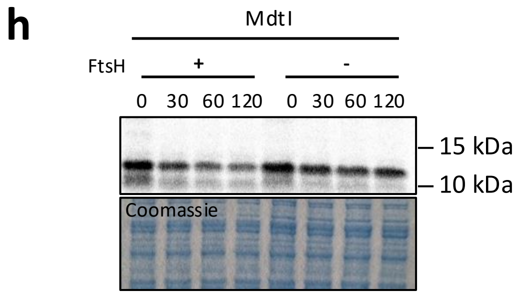

Supplementary figure 3. Effect of cytosolic stretches on the degradation of various proteins by FtsH (representative raw data for Fig. 4)

(a) Degradation of ASBT and its mutants in BL21 strain, assessed by radioactive pulse-chase.
(b) Quantification of (a). Shown are means ± SEM, quantified from at least three pulse-chase biological repeats. Solid lines depict nonlinear regression fits to an exponential decay equation where a fraction of the protein remains stable over time.
(c) Degradation of ASBT L73Q and its ΔN mutant in the presence and absence of FtsH.
(d) Degradation of truncation mutants where the loop between GFP and MdtJ of the C-terminal tail is shortened, as probed by translation shutoff and visualization of the degradation of the fluorescent GFP-fused protein.
(e) A table showing the GFP-MdtJ loops sequences before and after mutagenesis.
(f) Degradation of MdtJ without the N and C tail, probed by radioactive pulse-chase.
(g) A table showing the MdtJ loops sequences before and after mutagenesis.
(h) Degradation of MdtI, as probed by radioactive pulse-chase, in the presence and absence of FtsH.

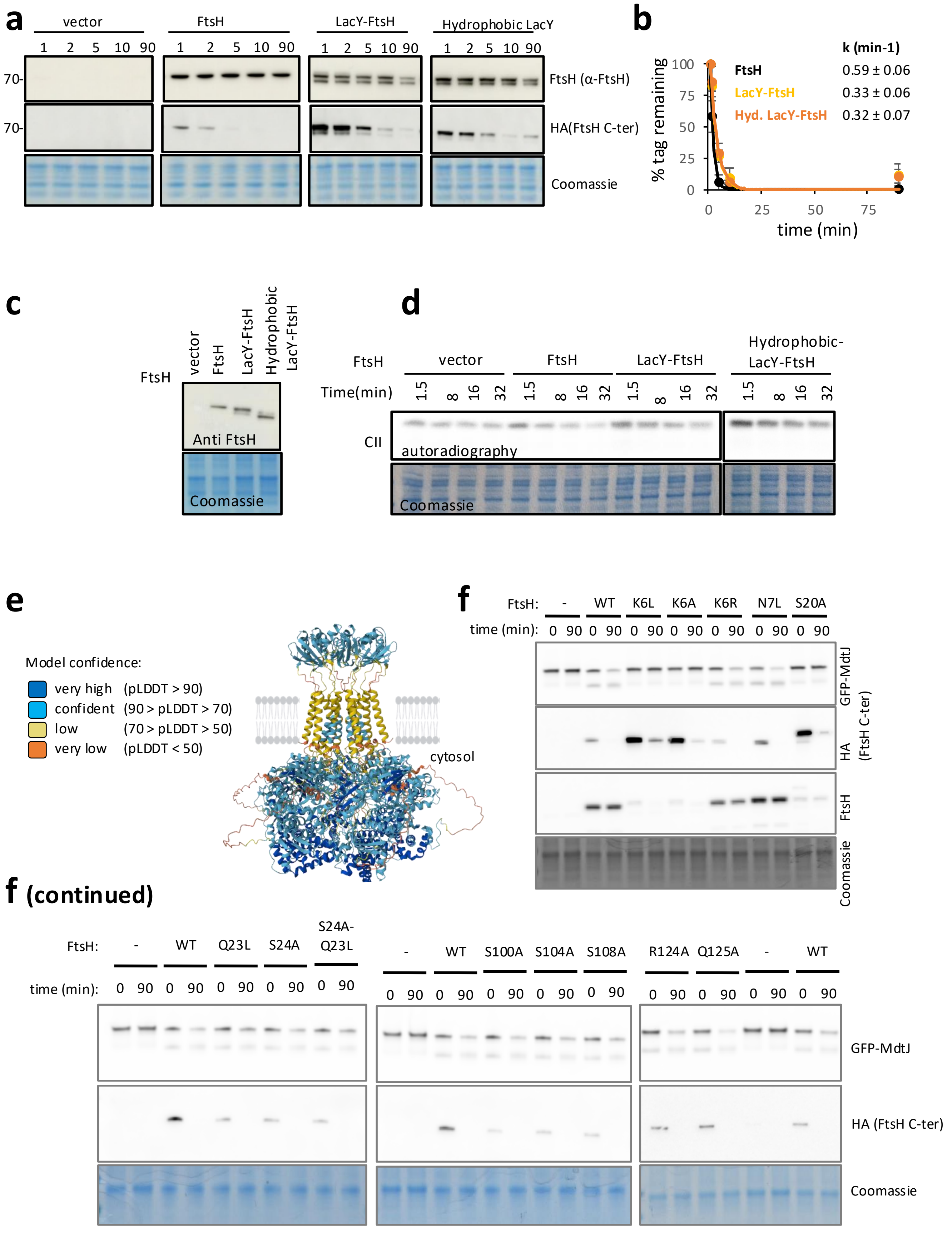

Supplementary figure 4, panels a-f. Effects of mutating the transmembrane domain of FtsH (representative raw data for Fig. 5,6).

(a) Representative Western blots showing the kinetics of LacY-FtsH chimeras degradation of the FtsH C-terminal HA tag. The degradation experiment was done similarly to Fig. 5c, with additional detection of FtsH C-terminus by anti-HA antibodies. Coomassie is shown as a loading control.

(b) The self-cleavage of LacY-FtsH chimeras is kinetically comparable to the WT. The amount of HA-reactive FtsH was quantified using densitometry. The kinetics were fitted to the formula $Ct = C0 \times e{-}kt$ (line). $C0$, initial amount of FtsH at time t=0; k, rate constant. The obtained values of k are shown in the inset. The amount of FtsH at time zero was omitted from fitting to reduce error.

(c,d) LacY-FtsH chimeras degrade the cytosolic substrate CII. (c) Expression of the FtsH chimeras. (d) CII degradation was followed by radioactive pulse-chase followed by autoradiography. Coomassie is shown as a loading control.

(e) AlphaFold model of the FtsH hexamer (left), Confidence scoring suggests that the TM domain has limited accuracy.

(f) Representative gels showing the activity of FtsH mutants in degradation of the GFP-MdtJ and FtsH C-terminal HA tag. Coomassie is shown as a loading control. The S20A FtsH mutant also unexpectedly migrated more slowly in SDS-PAGE, suggesting an unexplained apparent mass shift (upper left, HA and FtsH panel).

g

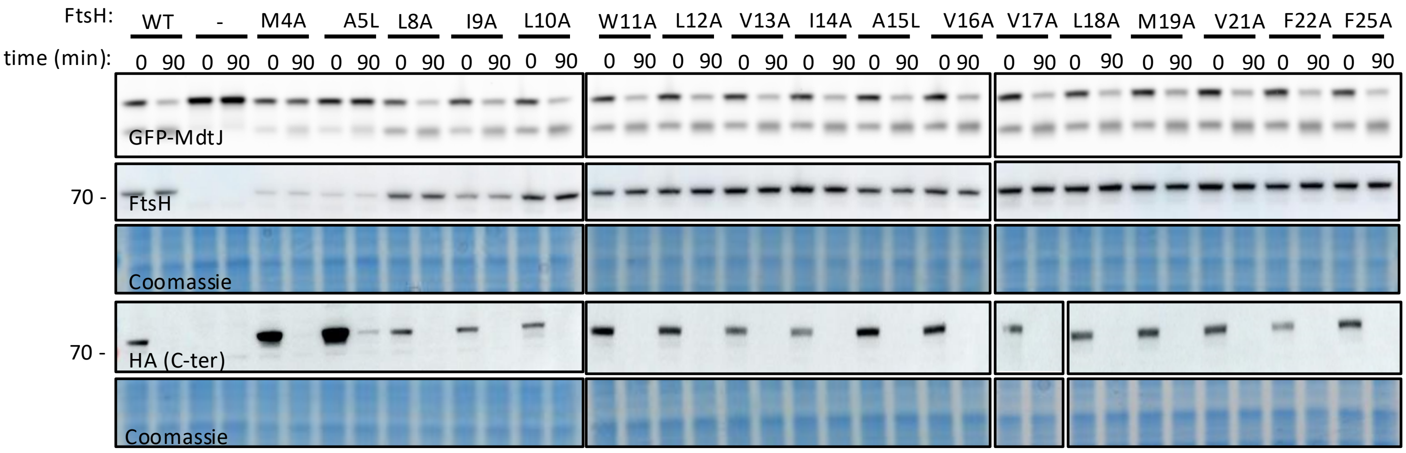

h

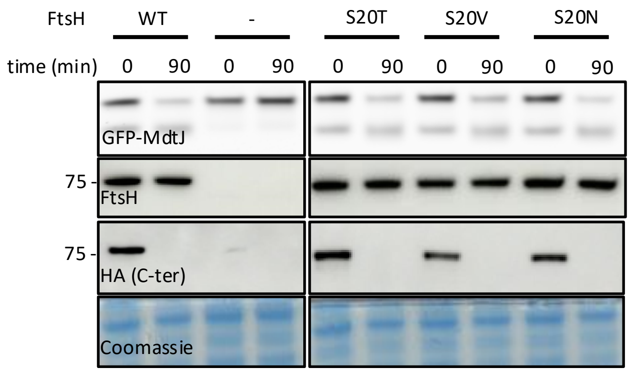

i

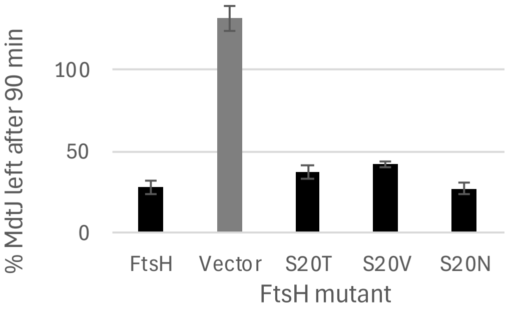

j

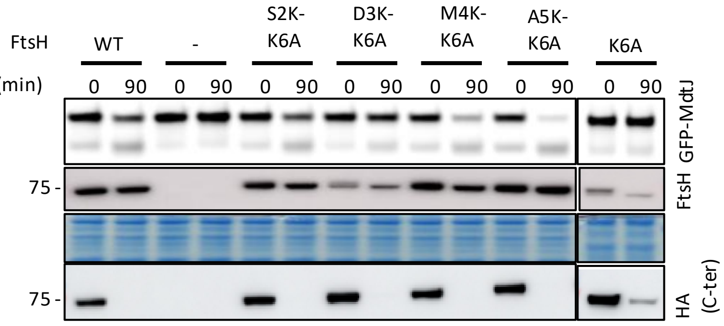

k

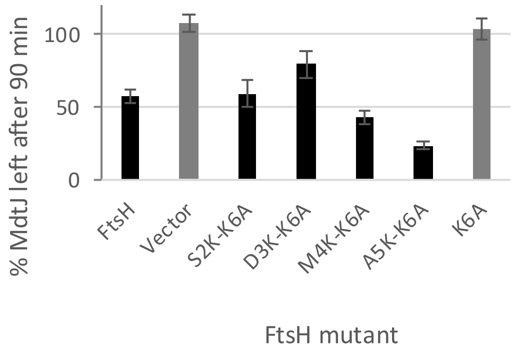

Supplementary figure 4 - continued.

(g) MdtJ degradation by apolar to alanine mutants in FtsH in the first TM helices. Most residues are dispensable for MdtJ degradation, except for mutations to M4, A5 that were impaired in expression.
(h,i) Activity of FtsH S20 residue variants in degradation of MdtJ and of the FtsH C-terminal HA tag. The degradation experiment was done similarly to Fig. 3d. (h) Representative gels show activity and expression of FtsH variants. The mutations did not affect FtsH self-cleavage activity. (i) Quantification of MdtJ degradation from h. Shown are averages ± SEM from three biological replicates.
(j,k) Activity of FtsH lysine variants, in degradation of MdtJ and of the FtsH C-terminal HA tag. The FtsH constructs were switched to plasmids with anhydrotetracycline (atc) induction for FtsH expression, in order to equalize the expression of FtsH variants. The degradation experiment was done similarly to Fig. 3d, with the addition of FtsH induction. K6A mutant was used as a control to show that even when induced, it does not degrade MdtJ. (j) Representative gels show activity and expression of FtsH variants. The mutations did not affect FtsH self-cleavage. (k) Quantification of MdtJ degradation from j. All mutants except D3K-K6A were able to degrade MdtJ. Shown are averages ± SEM from three biological replicates.

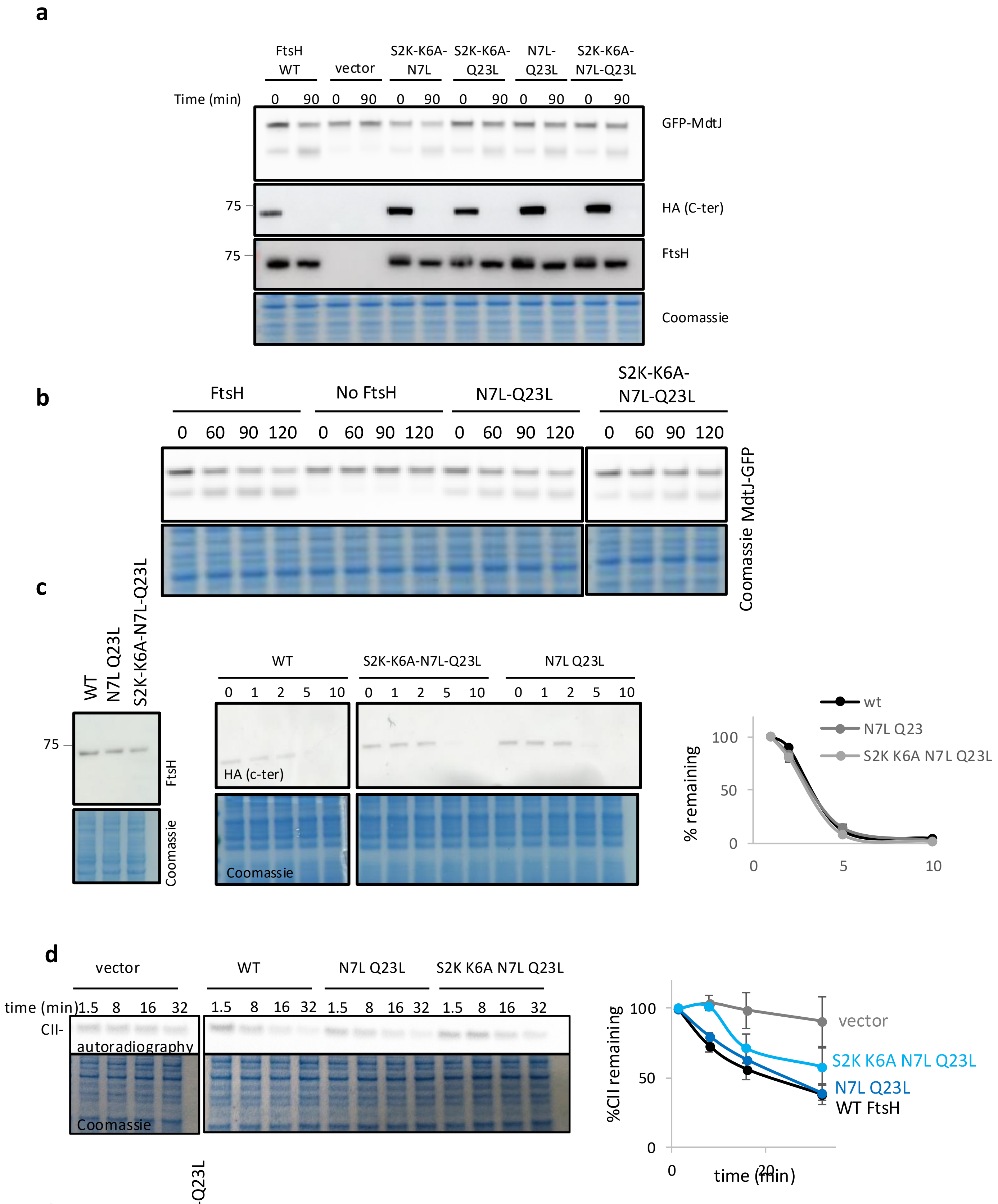

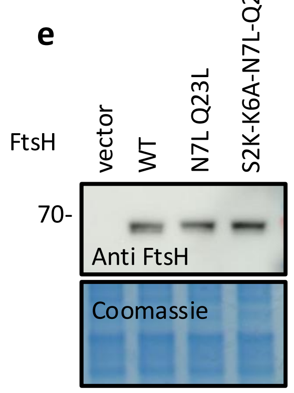

Supplementary figure 5. effects of mutations extending the TM1 hydrophobic core (representative raw data for Fig. 6).
FtsH constructs were switched to plasmids with anhydrotetracycline (atc) induction for FtsH expression, to equalize the expression of FtsH variants.
(a) Representative gels of the activity of FtsH variants in degradation of MdtJ. The degradation experiment was done similarly to Fig. 3d.
(b) Representative gel of degradation time course of GFP-MdtJ by FtsH TM1 variants, with polar to apolar mutations in the boundaries of TM1.
(c) The FtsH variants are active proteases, as observed by self-processing of the HA-tag. Left: expression of the FtsH variants. Middle: representative gel of degradation kinetics. Right: quantification of self-trimming kinetics, Shown are averages ± SEM from three biological replicates.
(d) The FtsH variants are active proteases, as observed by degradation of the cytosolic substrate CII. CII expression was followed by radioactive pulse-chase and autoradiography. Shown are averages ± SEM from three biological replicates.
(e) expression of the FtsH variants for experiment shown in d.

a

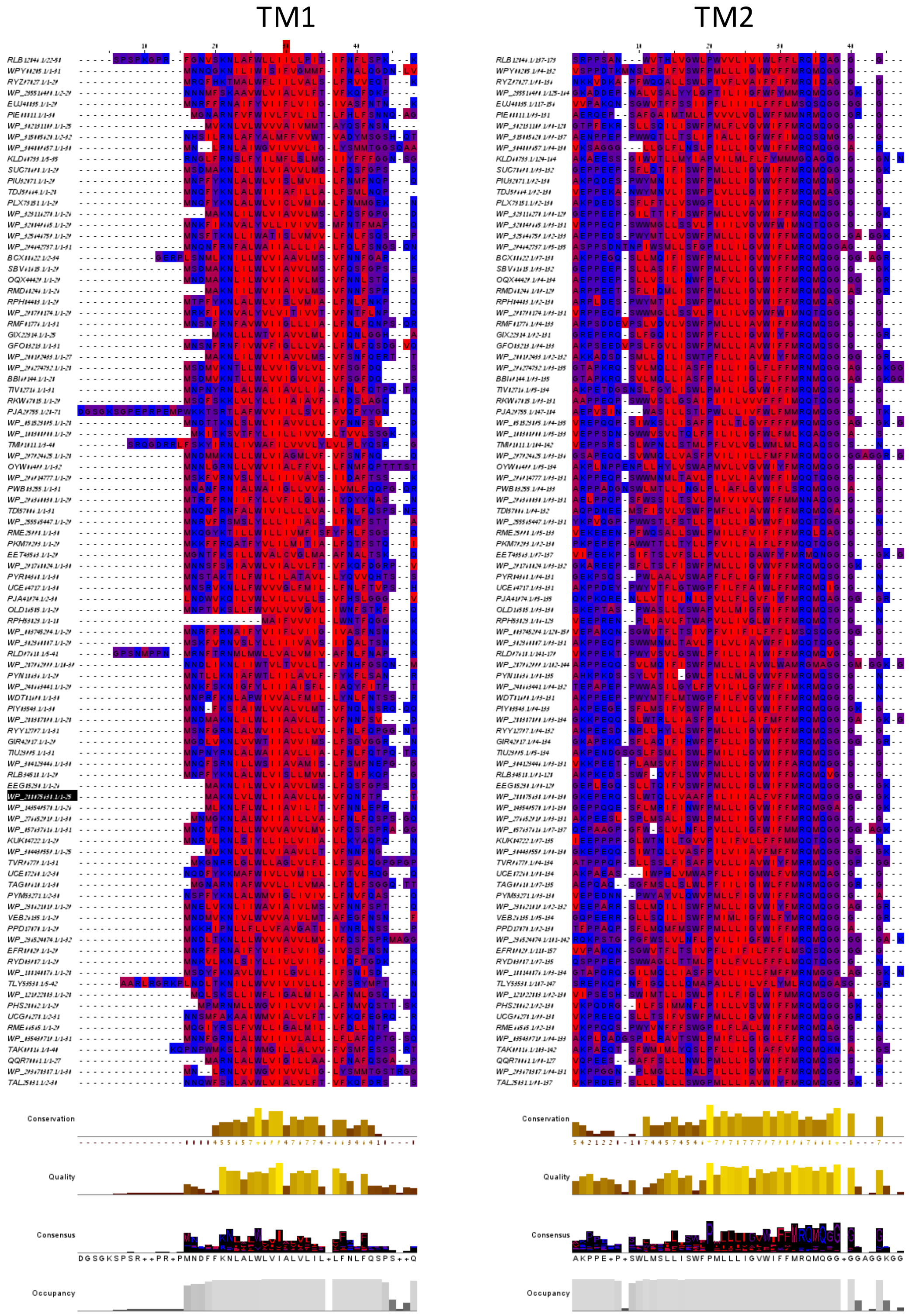

b

Organism	Protein	Localization	Sequence	Hydrophobic Length (aa)
E.coli	FtsH	Plasma membrane	SDMA**KNLILWLVIAVVLMSVFQ**SFGPSES	15
Bacillus subtilis	FtsH	Plasma membrane	NRVF**RNTIFYLLILLVVIGVVSYFQ**TSNP	18
Thermotoga maritima	FtsH	Plasma membrane	RSNIW**NLLFTILIIVTLFWLAR**FFYVENSP	15
Aquifex aeolicus	FtsH	Plasma membrane	MNAL**KNFFIWAIIGAAIVAFN**LFEGKREF	15
Pseudomonas putida	FtsH	Plasma membrane	AMNK**DKPWTLYLLAVGLAVLAAVQ**FGLFSQ	17
Saccharomyces cerevisiae	YME1 (i-AAA)	Mitochondria	FTVVSRWV**KWLLVFGILTYSFSE**GFK	13
Saccharomyces cerevisiae	YTA12 (m-AAA)	Mitochondria	NPVSKNVNLF**QIGLTFFLLSFLLD**LLN	12
Homo sapiens	YME1L1 (i-AAA)	Mitochondria	SLRRT**RLILFVLLLFGIYGLLKN**PFLSVRFRT	15

Supplementary figure 6. Sequences of FtsH TMs across organisms.

(a) Multiple sequence alignment of FtsH TMs 1 and 2, across 94 nonredundant bacterial homologs. The Multiple sequence alignment (Materials and Methods), viewed by Jalview, is colored by hydrophobicity (red- hydrophobic, blue- hydrophilic).

(b) Several FtsH homologues from model organisms display the feature of a short hydrophobic stretch in TM1. The hydrophobic stretch (black), along with flanking highly polar residues (color), is highlighted in bold.

Supplementary Table 1. List of plasmids used

Purpose	Plasmids
**ASBT purification,**<br><br>**ASBT radioactive pulse-chase degradation**	pZ-ASBT WT<br>pZ-ASBT L73Q<br>pZ-ASBT L73A<br>pZ-ASBT L73S<br>pZ-ASBT L73E<br>pZ-ASBT L73K<br>pZ-ASBT ΔTM10<br>pZ-ASBT WT ΔN tail<br>pZ-ASBT L73Q ΔN tail
**ASBT crosslinking with dBBr**	pZ- WT ASBT<br>pZ-ASBT 92C<br>pZ- ASBT 219C<br>pZ- ASBT 92C 219C<br>pZ- ASBT L73Q 92C 219C<br>pZ-ASBT ΔTM10 92C 219C
**GFP-MdtJ degradation**	pBAD sfGFP STD MdtJ<br>pBAD sfGFP STD MdtJ E23L<br>pBAD sfGFP STD MdtJ T27V S28A<br>pBAD sfGFP STD MdtJ S52A<br>pBAD sfGFP STD MdtJ S56A<br>pBAD sfGFP STD MdtJ Y57F Y58F<br>pBAD sfGFP STD MdtJ E77L<br>pBAD sfGFP STD MdtJ T85V<br>pBAD sfGFP STD MdtJ R100A<br>pBAD sfGFP STD MdtJ S103A<br>pBAD sfGFP STD MdtJ S109A<br>pBAD sfGFP STD MdtJ S56A E77L<br>pBAD STD GFP- MdtJ + MdtI<br>pBAD STD GFP- MdtJ L55Q<br>pBAD STD GFP- MdtJ L79Q<br>pBAD sfGFP STD MdtJ Δ17<br>pBAD sfGFP STD MdtJ Δ17 Δtail
**MdtJ degradation**	pET19b- MdtJ<br>pET19b - MdtJ ΔN tail<br>pET19b - MdtJ ΔC tail<br>pET19b - MdtJ ΔN,C tail<br>pET19b - MdtI
**MdtJI toxicity assay**	pET19b<br>pET19b MdtJ<br>pET19b MdtJ+MdtI<br>pET19b MdtJ S56A E77L

Purpose	Plasmids
**FtsH plasmids**	pACYC vector<br>pACYC FtsH 3HA tag<br>pACYC FtsH H417Y 3HA tag<br>pACYC FtsH K6A 3HA tag<br>pACYC FtsH K6L 3HA tag<br>pACYC FtsH K6R 3HA tag<br>pACYC FtsH N7L 3HA tag<br>pACYC FtsH S20A 3HA tag<br>pACYC FtsH Q23L 3HA tag<br>pACYC FtsH S24A 3HA tag<br>pACYC FtsH S100A 3HA tag<br>pACYC FtsH S104A 3HA tag<br>pACYC FtsH S108A 3HA tag<br>pACYC FtsH R124A 3HA tag<br>pACYC FtsH Q125L 3HA tag<br>pACYC FtsH Q23LS24A -3HA<br>pACYC FtsH S100AS104A -3HA<br>pACYC FtsH S104AS108A -3HA<br>pACYC lacY-FtsH-3HA<br>pACYC hydrophobic lacY-FtsH-3HA<br>pACYC FtsH M4A 3HA<br>pACYC FtsH A5L 3HA<br>pACYC FtsH L8A 3HA<br>pACYC FtsH I9A 3HA<br>pACYC FtsH L10A 3HA<br>pACYC FtsH W11A 3HA<br>pACYC FtsH L12A 3HA 3HA<br>pACYC FtsH V13A 3HA<br>pACYC FtsH I14A 3HA<br>pACYC FtsH A15L 3HA<br>pACYC FtsH V16A 3HA<br>pACYC FtsH V17A 3HA<br>pACYC FtsH L18A 3HA<br>pACYC FtsH M19A 3HA<br>pACYC FtsH V21A 3HA<br>pACYC FtsH F22A 3HA<br>pACYC FtsH S20T 3HA<br>pACYC FtsH S20V 3HA<br>pACYC FtsH S20N 3HA
**FtsH plasmids under tetracycline control**	pACYC tet R tetP FtsH S2K-K6A 3HA<br>pACYC tet R tetP FtsH D3K-K6A 3HA<br>pACYC tet R tetP FtsH M4K-K6A 3HA<br>pACYC tet R tetP FtsH A5K-K6A 3HA<br>pACYC tet R tetP FtsH S2K-K6A-N7L 3HA<br>pACYC tet R tetP FtsH S2K-K6A-Q23L 3HA<br>pACYC tet R tetP FtsH N7L-Q23L 3HA<br>pACYC tet R tetP FtsH S2K-K6A-N7L-Q23L 3HA
**cII degradation assay**	pZ- cII
